# A Position Effect on the Heritability of Epigenetic Silencing

**DOI:** 10.1371/journal.pgen.1000216

**Published:** 2008-10-10

**Authors:** Jaswinder Singh, Michael Freeling, Damon Lisch

**Affiliations:** 1 Plant Science Department, McGill University, Macdonald Campus, Ste. Anne de Bellevue, Quebec, Canada; 2 Department of Plant and Microbial Biology, University of California Berkeley, Berkeley, California, United States of America; Fred Hutchinson Cancer Research Center, United States of America

## Abstract

In animals and yeast, position effects have been well documented. In animals, the best example of this process is Position Effect Variegation (PEV) in *Drosophila melanogaster*. In PEV, when genes are moved into close proximity to constitutive heterochromatin, their expression can become unstable, resulting in variegated patches of gene expression. This process is regulated by a variety of proteins implicated in both chromatin remodeling and RNAi-based silencing. A similar phenomenon is observed when transgenes are inserted into heterochromatic regions in fission yeast. In contrast, there are few examples of position effects in plants, and there are no documented examples in either plants or animals for positions that are associated with the reversal of previously established silenced states. *MuDR* transposons in maize can be heritably silenced by a naturally occurring rearranged version of *MuDR*. This element, *Muk*, produces a long hairpin RNA molecule that can trigger DNA methylation and heritable silencing of one or many *MuDR* elements. In most cases, *MuDR* elements remain inactive even after *Muk* segregates away. Thus, *Muk*-induced silencing involves a directed and heritable change in gene activity in the absence of changes in DNA sequence. Using classical genetic analysis, we have identified an exceptional position at which *MuDR* element silencing is unstable. *Muk* effectively silences the *MuDR* element at this position. However, after *Muk* is segregated away, element activity is restored. This restoration is accompanied by a reversal of DNA methylation. To our knowledge, this is the first documented example of a position effect that is associated with the reversal of epigenetic silencing. This observation suggests that there are *cis*-acting sequences that alter the propensity of an epigenetically silenced gene to remain inactive. This raises the interesting possibility that an important feature of local chromatin environments may be the capacity to erase previously established epigenetic marks.

## Author Summary

Epigenetics involves the heritable alteration of gene activity without changes in DNA sequence. Although clearly a repository for heritable information, what makes epigenetic states distinct is that they are far more labile than those associated with DNA sequence. The epigenetic landscape of eukaryotic genomes is far from uniform. Vast stretches of them are effectively epigenetically silenced, while other regions are largely active. The experiments described here suggest that the propensity to maintain heritable epigenetic states can vary depending on position within the genome. Because transposable elements, or transposons, move from place to place within the genome, they make an ideal probe for differences in epigenetic states at various positions. Our model system uses a single transposon, *MuDR* in maize, and a variant of *MuDR*, *Mu killer* (*Muk*). When *MuDR* and *Muk* are combined genetically, *MuDR* elements become epigenetically silenced, and they generally remain so even after *Muk* is lost in subsequent generations. However, we have identified a particular position at which the *MuDR* element reactivates after *Muk* is lost. These data show that there are some parts of the maize genome that are either competent to erase epigenetic silencing or are incapable of maintaining it. These results suggest that erasure of heritable information may be an important component of epigenetic regulation.

## Introduction

Whether or not a gene is expressed can depend as much on its location within the genome as its primary DNA sequence. Although proximity to enhancers and suppressors outside the core promoter can affect gene expression, the most dramatic position effects often involve epigenetic silencing of genes placed in proximity to inactive or heterochromatic regions of the genome. In animals, the best example of this process is Position Effect Variegation (PEV) in *Drosophila melanogaster* [1],[2]. In PEV, when genes are moved into close proximity to constitutive heterochromatin, their activity can become unstable, resulting in variegated patches of gene expression. This process is regulated by a variety of proteins implicated in both chromatin remodeling [3]–[5] and RNAi-based silencing [6]. PEV appears to be the result of the spreading of a compacted chromatin state from heterochromatin to adjacent genes. Given that heterochromatin is largely composed of transposable elements, PEV can be seen as a breakdown in the normal process by which transposable elements and host genes are effectively sequestered from each other. The spread of heterochromatin can be blocked by insulating sites, such as those bound by Suppressor of Hairy-wing [7],[8] and GAGA factor [9],[10]. These proteins are competent to alter the silenced state by actively remodeling chromatin. Interestingly, some of the same proteins, such as GAGA factor, are also involved in the epigenetic regulation of homeobox genes during *Drosophila* development. These observations suggest that the process by which transposable elements are sequestered from the rest of the genome may have been recruited to regulate host gene expression as well.

Phenomena similar to PEV have also been observed in *Schizosaccharomyces pombe*. In this case, transgenes integrated into centromeric heterochromatin or silent mating type loci become silenced [1]. Many of the proteins that have been identified that influence this process are conserved among eukaryotes [11], and can affect gene silencing in species as diverse as *Arabidopsis thaliana* [12] and humans [13]. A number of proteins that influence centromeric silencing in *S. pombe* have orthologs in *Drosophila* that regulate PEV [11]. Thus, there are clear and consistent relationships between position effects, chromatin structure and epigenetic silencing.

Although a great deal is known about position effects in *Drosophila* and fission yeast, very little is known about it in plants. Indeed, there is conflicting evidence as to whether or not they exist at all in plants [14]–[16]. Certainly, transgenes equipped with minimal promoters can respond to local tissue-specific enhancers [17], but position-specific effects on the epigenetic state of genes, such as has been observed in *Drosophila* and yeast, have not been well documented. In plants, variations in expression of transgenes at various locations have been interpreted as “position effects”. However, the stochastic nature of transgene silencing, variations in copy number and sequence of integrated transgenes and sporadic tissue-culture induced epigenetic variation make interpretation of these experiments difficult.

Ideally, to prove a position effect, the effect should be reversible due to subsequent changes in position. Since transposable elements are mobile, they represent an ideal model for understanding the role of position in gene activity. Among transposable elements, the *Mutator* (*Mu*) transposons in maize are particularly useful because they transpose at a high frequency and can be epigenetically silenced in a controlled fashion [18]. *Mutator* is the most active known plant transposon. In *Mu*-active lines, *Mu* elements can duplicate at a 100% frequency; every element makes an average of one duplication every generation [19]. Insertions are into unlinked sites, and the overall mutation frequency in an active line can exceed 50 times that of background [20]. The system is regulated by *MuDR* elements, which carry two genes: *mudrA* and *mudrB* [18]. These genes encode MURA, the putative transposase, and MURB, a helper protein of unknown function. We have derived a minimal version of this transposon system, that includes a single active *MuDR* element and a single non-autonomous reporter element inserted into a color gene [21]. In the presence of an active *MuDR* element, the non-autonomous element excises from the color gene during somatic development, resulting in small sectors of revertant tissue. Unlike higher copy number *Mu* lines, the minimal line does not undergo spontaneous silencing. However, a single derivative of *MuDR* arose in the minimal line that can heritably silence one or many *MuDR* elements [22]. This derivative, called *Mu killer (Muk)*, contains a portion of *MuDR* that has been duplicated and inverted. The *Muk* transcript forms a perfect 2.4 kb hairpin RNA, which is processed into 26 nt siRNAs [23]. These siRNAs trigger rapid degradation of the *mudrA* transcript, as well as methylation of the terminal inverted repeats (TIRs) and transcriptional silencing of one or many *MuDR* elements. After exposure to *Muk*, *MuDR* elements generally remain heritably and stably silenced even in the absence of *Muk*. The availability of the *Muk* locus has made it possible to target *MuDR* elements for heritable epigenetic silencing in a controlled and reproducible fashion by making the appropriate genetic crosses.

The minimal *Mutator* line began with a single active *MuDR* element that can move from place to place in the genome. It was therefore possible to examine the effects of *Muk* on duplicate copies of the same *MuDR* element at various positions. Given that *Muk*-mediated silencing of *MuDR* involves *trans*-acting siRNAs, it seemed likely that, regardless of position, all *MuDR* elements would be silenced in the presence of *Muk*. In fact, we have found that silencing is particularly effective when multiple *MuDR* elements are present (Slotkin and Lisch, unpublished data). However, it was also possible that the degree to which individual elements would *remain* heritably silenced in the absence of *Muk* might vary depending on the local context. A screen was developed that made it possible to isolate individual duplications of a single active *MuDR* element, expose them to *Muk*, and observe the degree of heritable activity in progeny plants that carried the transposed copies of *MuDR* but that lacked *Muk*. This screen lead to the identification of a *MuDR* element at a specific chromosomal location that failed to maintain a heritable silenced state. We suggest that this phenomenon represents the converse of PEV, in that *cis* acting sequences in this case are responsible for reversing, as opposed to triggering, epigenetic silencing. The existence of such a locus suggests that an important feature of the epigenome may be the capacity to reverse epigenetic silencing.

## Materials and Methods

### *MuDR* Terminology

All *MuDR* elements described in this manuscript were derived from a single *MuDR* element that had been genetically isolated and cloned previously [21]. We have found that there are variations in duplication frequency and somatic activity depending on the position of transposed copies of this element [24]. Therefore, the elements at various positions are given distinct position numbers, indicated by parentheses. Thus, the original element is designated *MuDR(p1)* and duplicates are given new position numbers as they are characterized.

### Maize Stocks

**Figure 1:**
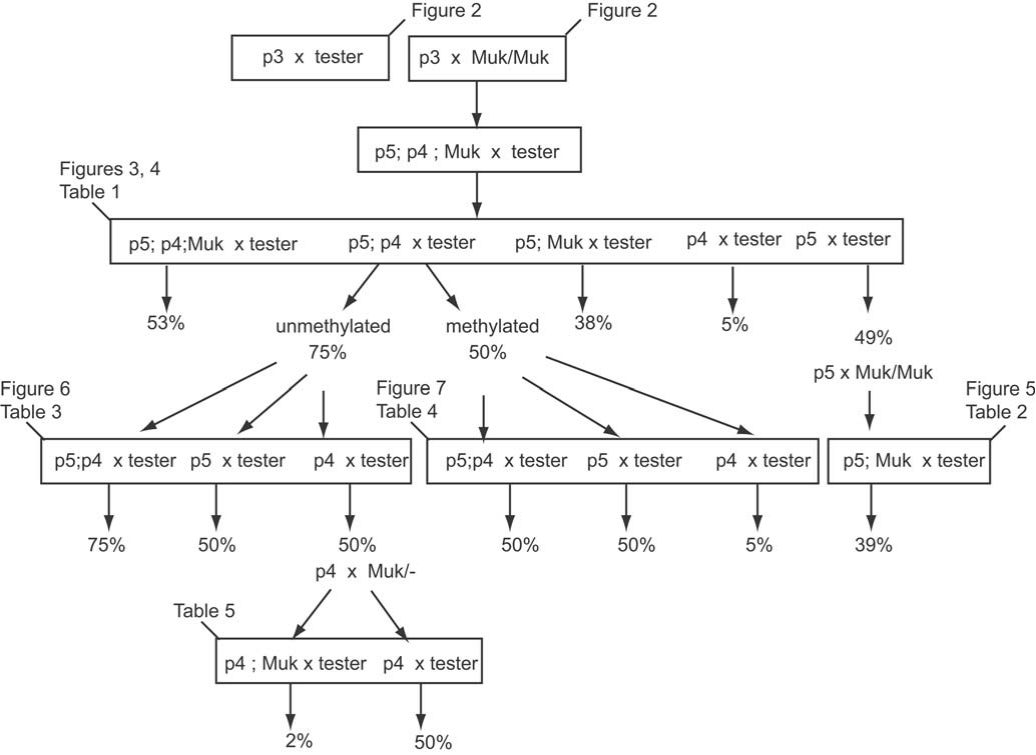
The crossing schemes used to generate the families described in the text. Tables and figures referring to particular families are as indicated. “p5” refers to *MuDR(p5)*; “p4” refers to *MuDR(p4)*. Percentages refer to the percent of spotted progeny kernels arising from a given cross.

**Figure 2:**
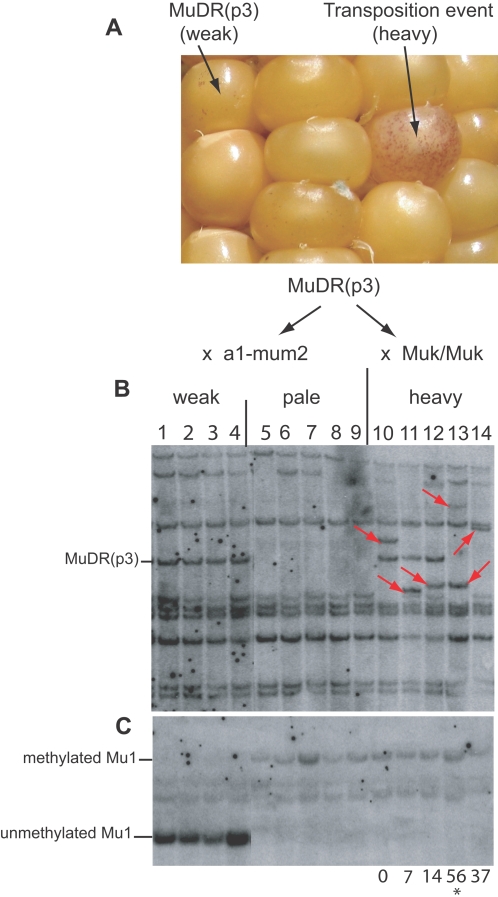
An ear derived from a plant carrying *MuDR(p3)* and Southern blot of DNA from plants grown from the test cross and the cross to a *Muk* homozygote. A) An ear derived from a plant carrying *MuDR(p3)* that was crossed as a female to a plant that was homozygous for *Muk*. Because *Muk* does not alter somatic excision frequency in F1 aleurone, changes in excision frequency from low to high could be used to screen for new insertions of *MuDR(p3)*, as is indicated. Kernels from this ear and the control test cross ear (*MuDR(p3*)×*a1-mum2* tester, not shown) were separated by excision frequency and planted. B) Southern blot of DNA from plants grown from the test cross (lanes 1–9) and the cross to a *Muk* homozygote (lanes 11–14). In the top panel, the DNA was digested with *Eco*RI, used to distinguish *MuDR* elements at different positions based on size polymorphisms, and probed with an internal fragment of *MuDR* (probe B, Figure 3). The red arrows indicate new MuDR insertions. In the bottom panel, the DNA was digested with the methyl-sensitive enzyme *Hin*fI and probed with an internal portion of *Mu1* (probe C, Figure 3). The resulting fragments resulting from methylated and unmethylated *Hin*fI sites in the end of the *Mu1* element at the *a1-mum2* reporter are as indicated. Following analysis of the DNA, each plant was then crossed to an *a1-mum2* tester. The numbers below the blots indicate the percent frequency of spotted kernels arising from test crosses of plants in the lanes above them.

The derivation of all families described in this manuscript is shown in Figure 1. This diagram follows standard conventions. Females are on the left and males on the right of the “x”. Unlinked loci are separated by a semicolon. *MuDR* elements at each position (designated “p4” or “p5”) are hemizygous for the insertion. All *MuDR* elements described here are derived from a single *MuDR* element originally present in the minimal *Mutator* line. The derivation of the minimal line, containing a single *MuDR* element and a single *Mu1* element inserted into the *a1-mum2* allele of the *A1* gene was described in Chomet et al. [21]. In the presence of active *MuDR* elements, *Mu1* excises from the *a1-mum2* allele, resulting in characteristically small revertant sectors (spots). These sectors are most readily visualized in the outer layer of the kernel (the aleurone). In the absence of *MuDR*, the reporter element remains inserted in the *A1* gene, and the kernels are uniformly colorless, or pale. The *a1-mum2* allele has the additional advantage of being suppressible in the adult tissues (but not in the kernel). In the adult tissues, expression of a functional gene product from *a1-mum2* is prevented by the presence of *MuDR* transposase (MURA), except when *Mu1* excises from the allele. This results in characteristically small red (revertant) spots of color on a green (suppressed) background. When the transposase is lost, the adult tissue is uniformly red because the *a1-mum2* allele expresses in its absence [21]. This characteristic makes it possible to assay for transposase activity in mature plant tissue. In contrast, the aleurone layer of the kernels, *a1-mum2* is not suppressible. Thus, in the absence of the transposase, the kernels are uniformly pale, as can be seen in Figure 2A. All individuals described in this work were homozygous for the *a1-mum2* reporter allele. All crosses designated as “test crosses” represent crosses to the *a1-mum2* tester, which lacks both functional *MuDR* elements and *Mu killer*. The genetic isolation, characterization and cloning of *Muk* was described in Slotkin et al. [22],[23]. Genetic isolation and characterization of *MuDR(p3)* was described in Lisch et al. [24]. *MuDR(p3)* causes a distinctively low frequency of somatic excisions of *Mu1* from *a1-mum2* in the aleurone of the kernel. When *MuDR(p3)* transposes to a new position, somatic excision returns to a more typical frequency. Thus, germinally transmitted transpositions of *MuDR* from position 3 to a more typical position can be detected as heavily spotted kernels in a family segregating for weakly spotted kernels. With respect to the crosses of *Muk* to plants carrying *MuDR(p3)*, previous work has demonstrated that, when *Muk* is used as a male parent there is little or no effect on excision of the reporter *Mu1* element in the F1 aleurone, but a strong effect on *MuDR* elements in the F1 embryo and adult plant tissue [22]. Thus, transposed copies of *MuDR(p3)* can be easily detected as individual kernels with a high frequency of somatic excision of the reporter element in the aleurone, even when exposed to *Muk* derived from the male parent.

### DNA Extraction and Southern Blot Analysis

**Figure 3:**
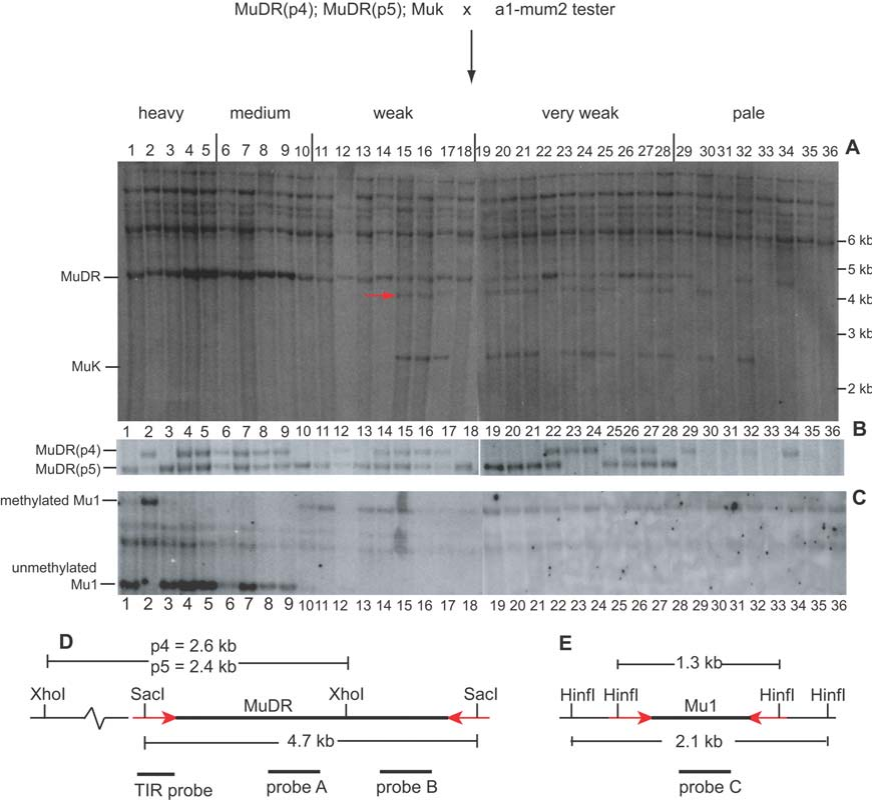
Southern blot analysis of a family segregating for *MuDR(p5)*, *MuDR(p4)* and *Muk*. Kernels were separated by somatic excision frequency and DNA was extracted from plants grown from those kernels. A) A *Sac*I digest probed with a fragment of *MuDR* (probe B). The diagnostic 4.9 kb *MuDR* fragment is as indicated. The smaller *Muk*-specific fragment is as indicated, as is as the larger fragment that results from methylation of the SacI site in the *Muk* TIR (red arrow). B) An *Xho*I digest of the same samples probed with a second fragment of *MuDR* (probe A). Polymorphisms specific to *MuDR* at each of two positions are as indicated. C) A *Hin*fI digest of the same samples probed with an internal fragment of *Mu1* (probe C). Fragments corresponding to unmethylated and methylated *Mu1* elements in this background are as indicated. D) A restriction map of *MuDR* with probe regions as indicated. The red arrows indicate TIRs. E) A restriction map of *Mu1* at *a1-mum2*.

DNA extraction and Southern blotting was as previously described [24]. Briefly, 10 micrograms of DNA was digested with a four-fold excess of restriction enzyme for a minimum of 2 hours, blotted and probed with a series of *Mu*-specific DNA fragments. Probes: The location of restriction enzyme sites and probes used are illustrated in Figure 3. The probes used to detect *MuDR* internal sequences (probes A and B) were as described in Slotkin et. al. [22]. The probed used for *Mu1* (probe C) was as described in Chomet et al. [21]. The probe for the *MuDR* TIR was generated by amplifying genomic DNA with primers TIRAF (GAGATAATTGCCATTATAGACGAAG) and TIRAR (AGGAGAGACGGTGACAAGAGGAGTA), which generates a fragment of 219 bp that includes the entire TIR (TIRA) flanking the *mudrA* gene of *MuDR*.

**Figure 4:**
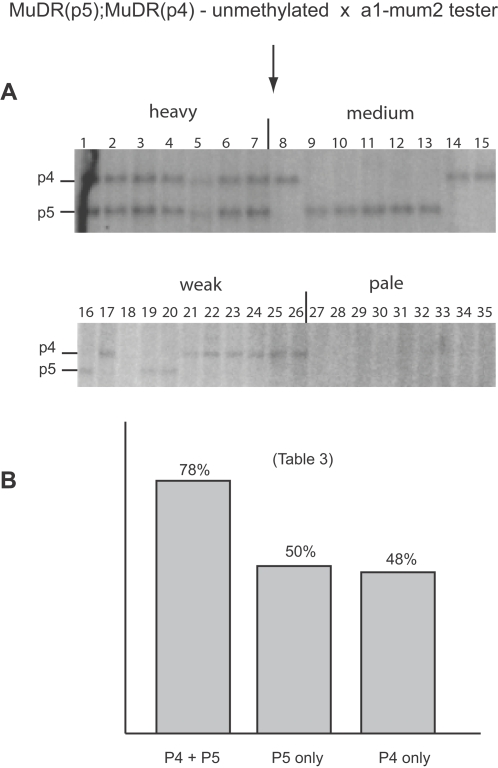
Genetic and Southern blot analysis of a family segregating for *MuDR(p5)*, *MuDR(p4)* and *Muk*. A) Graphic depiction of summarized frequency of spotted progeny kernels derived from different classes of individuals depicted in Figure 3. For each class, the relevant genotypes are as indicated. “meth” refers to the methylation status of *Mu1* elements of each class, as determined in Figure 3. B) Southern blot analysis of representative individuals from each class depicted in panel A. Samples were digested with *Hin*fI and probed with a fragment including all of the *MuDR* TIR. The relevant fragments are as indicated by the red arrows. The additional fragments visible on this blot represent *hMuDR* elements that do not cosegregate in this family with activity or a lack thereof. C) Restriction map of the region around one of the terminal inverted repeat flanking the *MuDR* elements. The indicated sizes are those expected if the *Hin*fI site in the TIR is methylated or unmethylated at the two positions based on available sequence. Because *Muk* has an identical TIR to *MuDR* and is methylated at the *Hin*fI, it can also be seen as a unique fragment of the indicated size. D) An example of a plant in which reactivation of *MuDR(p5)* was delayed. Because the reporter *a1-mum2* allele is suppressible, the green sectors represent tissue in which *MuDR(p5)* has been reactivated during somatic development.

Active *MuDR* elements, regardless of their position would be expected to yield a fragment of 445 bp when digested with *Hin*fI. This size is consistent with a lack of methylation of both the *Hin*fI site within the TIR adjoining *mudrA* (TIRA) of *MuDR* elements and of a second site within the first intron of *mudrA*. Methylation of the TIR *Hin*fI site of TIRA of *MuDR* elements will yield larger fragments whose size depends on the *MuDR* insertion sites. Based on the sequence of DNA flanking *MuDR(p4)* and *MuDR(p5)*, if the TIR *Hin*fI site (but not the internal *Hin*fI site) is methylated the expected fragment sizes are 648 bp and 1003 bp respectively. Similarly, the expected fragment size if the TIR *Hin*fI site is methylated in *Mu killer* is 500 bp. In each case the expected fragment sizes were observed (Figure 4B).

Hypomethylation of *Mu1 Hin*fI sites has proved to be a highly reliable indicator of MURA activity in our lines; the loss of *mudrA* transcript is invariably associated with methylation of this site [21],[24]. Methylation of *Mu1* elements was examined using *Hin*fI digests probed with an internal fragment of *Mu1*, as described in Chomet et al. [21]. An unmethylated *Mu1* element at *a1-mum2* is expected to give a fragment size of 1.3 kb; a methylated *Mu1* element at this locus gives a fragment size of 2.1 kb. In all cases, complete digestion of the DNA was confirmed by examination of the ethidium-stained gel.

To determine if full-length *MuDR* elements were present, *Sac*I was used. *MuDR* elements have two *Sac*I sites in the terminal inverted repeats (Figure 3). Digestion with this enzyme results in a diagnostic 4.7 kb fragment regardless of chromosomal position. The intensity of this fragment reflects the copy number of the element [24]. To detect transposed copies of *MuDR*, DNA samples were digested with *Eco*RI (Figure 2B) or *Xho*I (Figure 3B). These enzymes cut once within *MuDR*. Therefore, elements at various positions will give rise to unique fragment sizes.

### Cloning *MuDR(p4)* and *MuDR(p5)*

Cloning of these elements was achieved using inverse PCR. Southern blot analysis had revealed that digestion of samples containing these elements with *Xho*I yielded *MuDR* terminal inverted repeat (TIR)-hybridizing fragments of 2.6 and 2.4 kb for *MuDR(p4)* and *MuDR(p5)* respectively (Figure 2). 10 micrograms of DNA containing one or the other element was digested with a four-fold excess of *Xho*I for 4 hours in a total volume of 20 microliters. The reaction was placed at 65 degrees C for 15 minutes, to heat inactivate the restriction enzyme. Two microliters of the reaction was then added to 1 microliter of DNA ligase, two microliters of ligase buffer and 15 microliters of water, and the resulting mixture was incubated for 2 hours at 25 degrees C. The reaction was then heat inactivated for 15 minutes at 65 degrees C. Two microliters of this reaction was then subjected to PCR amplification using primers specific to the *MuDR* TIR (TIRout: GCTGTCACCTTTCTGTTTTGGCGAT) and a *MuDR* internal sequence flanking the *Xho*I site (exon3R: CTAGCTCTTGTTCAGTGACTTCC). These amplifications yielded products of 700 bp and 520 bp for samples containing *MuDR(p4)* and *MuDR(p5)* respectively, the expected sizes for these elements based on the *Xho*I restriction mapping data. Both strands of the PCR products were then sequenced using an ABI sequencer (Applied Biosystems). The sequences of *MuDR* obtained were identical to known *MuDR* sequences. The flanking sequences were used to design primers facing inwards towards the *MuDR* elements. These primers in combination with *MuDR* TIR primers were used to confirm that we had indeed cloned the elements. Flanking primers were used in combination with TIR-specific primers on DNA samples of plants segregating for each element. For *MuDR(p5)*, primer p5flnkB (CGATTAAGCGCGACGAACACG) was used in combination with RLTIR2 (ATGTCGACCCCTAGAGCA). In a family segregating for *MuDR(p5)* and *MuDR(p4)*, these primers gave a product of 408 bp in three of three plants carrying only *MuDR(p5)* and zero of three plants carrying only *MuDR(p4)*. To obtain sequences on the other side of the insertion, the available flanking sequences were used to search DNA databases for maize sequence matches. Perfect matches were used to extend the sequence, which were then used to design primers that would be expected to amplify when used in combination with a *MuDR* TIR primer. Primer p5flnkA (GGAGCGTGACAGGGGCGGCAGAT) was used with primer TIRAR (AGGAGAGACGGTGACAAGAGGAGTA). The same samples that yielded a product with the p5flnkB/RLTIR2 combination also yielded the expected 405 bp product. When the sequences of the DNA flanking the insertion were compared, they revealed the presence of a 9 bp target site duplication (GGCGTGCGC) diagnostic for *Mu* insertions. The strategy to confirm the *MuDR(p4)* was similar. The available sequence was used to design a flanking primer, p4flnkB (CGTGAAAGGTGGAGACTACTGGAA), which was used in combination with the *MuDR* TIRAR primer. A product of the expected size of 320 bp cosegregated with the presence of *MuDR(p4)*, confirming that we had also cloned sequences flanking *MuDR(p4)*.

## Results

### Transposed *MuDR* Elements Are Silenced by *Muk*

In order to screen for new insertions of single *MuDR* elements, we made use of a *MuDR* element that exhibits a position effect that results in reduced somatic excision of non-autonomous reporter element from a color gene in the aleurone (Figure 2A)(for alleles and stock construction see Materials and Methods). This effect on somatic excisions of the reporter is fully reversible; when *MuDR(p3)* transposes to a new position, the high frequency of excision and transposition more typical for *MuDR* are restored [24]. The advantage of using *MuDR(p3)* is that, in a family of kernels segregating for this element, new insertions of *MuDR(p3)* can be readily visualized as individual kernels exhibiting a high frequency of excisions. It should be emphasized that when *Muk* is introduced through the male lineage, it has no immediate effect on the F1aleurone, but it has a strong effect on the F1 embryo and the resulting plant [22]. Thus, individual kernels that inherit a transposed copy of *MuDR(p3)* would be expected to exhibit a high frequency of excision, even in the presence of *Muk*, but plants grown from those kernels would be expected to show reduced or absent *MuDR* activity.

To perform the screen, one ear of a plant carrying *MuDR(p3)* was crossed to the *a1-mum2* tester (the control cross), and a second ear from the same plant was crossed to a plant that was homozygous for *Muk*. These and subsequent crosses are portrayed in Figure 1. DNA from plants grown from weakly spotted and pale (non-spotted) sibling kernels derived from the control cross were examined by Southern blot for the presence of *MuDR(p3)*. As expected, all progeny plants grown from weakly spotted kernels carried the diagnostic 6.8 kb *MuDR(p3) Eco*RI fragment (Figure 2B, lanes 1–4). The other fragments hybridizing to this probe are inactive *MuDR* homologs (*hMuDR*s) that do not positively or negatively affect *Mu* activity in this line [24],[25]. Methylation of *Mu1* at *a1-mum2* was also assayed because *Mu1* methylation has proved to be a highly reliable indicator of *MuDR* activity. The *Mu1* elements in the individuals carrying *MuDR(p3)* were unmethylated due to the presence of the *MuDR(p3)*-derived transposase (Figure 2C, lanes 1–4). Sibling plants grown from non-spotted kernels that did not inherit *MuDR(p3)* (lanes 5–9) carried methylated *Mu1* elements, a consequence of the absence of a functional *MuDR* element.

In the experimental cross (*MuDR(p3)/- x Muk/Muk*), only plants grown from heavily spotted kernels (which were expected to contain duplicate copies of *MuDR(p3)*), were examined (Figure 2A, lanes 10–14). In each case, an *Eco*RI digest revealed that plants grown from these kernels contained at least one new *MuDR* insertion (red arrows in Figure 2B). *Mu* elements transpose duplicatively in the germinal lineage [24]. Therefore, the absence of *MuDR(p3)* in plants grown from some of the heavily spotted kernels was not due to germinally transmitted excisions of *MuDR(p3)*. *Mu* elements do, however, often transpose just prior to meiosis. Thus some of these plants carried *MuDR(p3)*, while others carried only transposed copies of that element due to independent assortment of the donor and transposed elements. Previous work in our laboratory has demonstrated that although *Muk* has no effect on *MuDR* activity in the aleurone if *Muk* is introduced via the male parent, it has a strong effect on *MuDR* activity in the F1 embryo and plant [22]. This was observed in the plants grown from the heavily spotted kernels that carried transposed copies of *MuDR(p3)*. Each plant contained *Mu1* elements that were methylated, consistent with the loss of transposase in these plants due to the activity of *Muk* [22] (Figure 2C, lanes 10–14). As described in Materials and Methods, the *a1-mum2* allele is suppressible in the adult plant tissue, resulting in red plants in the absence of *MuDR* activity and green plants with small revertant sectors in its presence [21]. This made it possible to monitor activity by observing plant color. All of the plants carrying *Muk* in this experiment were red, consistent with the loss of *MuDR* activity. We conclude from this experiment that each of these plants contained at least one newly transposed copy of *MuDR(p3)*, and that *Muk* was efficiently silencing all of these elements.

### A Transposed Element Becomes Reactivated after the Loss of *Muk*

To test for heritability of silencing, each plant carrying a transposed copy of *MuDR(p3)* was crossed as a female to the *a1-mum2* tester. Typically, the ears resulting from a cross of a plant carrying both *Muk* and one or many *MuDR* will exhibit a low frequency of spotted progeny kernels, most of which are only weakly spotted [22]. This was true for three of the five individuals examined, and these results are consistent with heritable silencing of transposed *MuDR* elements in these plants. A fourth plant gave rise to a higher overall percent of spotted progeny (37%), but these kernels were uniformly weakly spotted, and this family was not examined further. In contrast, one plant gave rise to an ear with an unusually high proportion of heavily spotted kernels (Figure 2B, lane 13). Overall, the family derived from the test cross of this plant had 57% (83/147) spotted progeny kernels, roughly half of which (46/83) were more heavily spotted. This plant lacked *MuDR(p3)* and contained two new *MuDR*-hybridizing fragments, which we designated *MuDR(p4)* and *MuDR(p5)*. Progeny kernels were separated into classes based on excision frequency, with the expectation that excision frequency would reflect the degree of heritable activity. The more heavily spotted kernels are designated “heavy” and “medium” in Figure 3. Plants grown from representatives of each excision frequency class were then subjected to Southern blot analysis (Figure 3).

In order to detect the presence of full-length transposed *MuDR* elements, a *Sac*I digest of DNA from this family probed with a fragment of *MuDR* was compared to an *Xho*I digest, also probed with *MuDR*. *Sac*I cuts in the ends of *MuDR* and gives rise to a diagnostic 4.7 kb fragment regardless of the element's position; in this genetic background only full-length functional *MuDR* elements yield a fragment of this size [24]. Because *Muk* has sequence identity to *MuDR* in the probe region [23], this derivative of *MuDR* can also be observed as a 2.5 kb fragment (i.e. lanes 15,16 and 17 in Figure 3A). *Sac*I sites in *Muk* are subject to partial methylation (Slotkin and Lisch, unpublished data), resulting in the larger, 4.2 kb fragment in plants with *Muk* as well (red arrow, Figure 3A).

*Xho*I cuts only once in *MuDR*. Therefore, elements at various chromosomal positions give rise to unique fragment sizes (Figures 3B and 3D). Our analysis revealed that each of two *Xho*I segregating fragments contributed to the intensity of the *Sac*I internal fragment. When both *Xho*I fragments were missing, so was the diagnostic *Sac*I fragment. All spotted kernels gave rise to plants with one or the other *Xho*I fragment; kernels that lacked both *Xho*I fragments (Figure 3, lanes 30, 31, 33, 35 and 36) were uniformly pale and did not transmit spotted progeny kernels when plants grown from those kernels were test crossed. We conclude from this analysis that each *Xho*I fragment represents a full length *MuDR* element that can condition somatic activity of the reporter. The element that gave the smaller *Xho*I polymorphism was arbitrarily designated *MuDR(p5)* and that which gave the larger polymorphism was designated *MuDR(p4)*.

The DNA samples from this family were also digested with *Hin*fI, the methyl-sensitive enzyme that cuts in the ends of the reporter *Mu1* element, and probed with *Mu1*. Strikingly, most (8/10) of the individuals grown from the most heavily spotted kernels contained unmethylated *Mu1* elements (Figure 3C). This reversal of *Mu* element methylation has not been observed before, and suggests that some feature of the *MuDR* elements in these plants had been altered. All plants that carried unmethylated *Mu1* elements carried *MuDR(p5)* and none of them carried *MuDR(p4)* by itself. None of these plants carried *Muk*, but 11/18 (61%) of plants grown from the more weakly spotted kernels did. None of the plants that were grown from weakly spotted kernels had hypomethylated *Mu1*. Overall, 26/28 (93%) of plants grown from kernels exhibiting any spotting at all carried *MuDR(p5)*. In contrast, only 17/28 (61%) of these plants carried *MuDR(p4)*, as did 3/8 (38%) of the plants grown from pale kernels. These results are consistent with segregation of a single active *MuDR* element (*MuDR(p5)*) and a second, much more weakly active element (*MuDR(p4)*). The presence of *Muk* in roughly half of plants grown from the weakly spotted kernels demonstrated that this locus had been in the parent and was still competent to silence *MuDR* elements.

Each plant from the above family was test crossed to determine the heritability of activity. The genetic ratios of spotted to pale kernels in the next generation were used to determine the copy number and degree of heritable activity of *MuDR* elements in each plant. The resulting families demonstrated an unambiguous relationship between *MuDR(p5)* and heritable activity as assayed by the number of spotted progeny kernels from these test crosses. Plants carrying only *MuDR(p5)* and unmethylated *Mu1* elements gave rise to an average of 50% spotted kernels, consistent with segregation of a single, fully active *MuDR* element (Figure 4A and Table 1). Many of the plants examined that lacked *Muk* and that carried *MuDR(p5)* carried methylated *Mu1* elements in the first generation following the loss of *Muk*. This suggested that in the leaf tissue of these plants, *MuDR* remained inactive. However, these plants exhibited a sectored phenotype with respect to expression of the suppressible *a1-mum2* allele in the first generation following the loss of *Muk* (Figure 4D). This phenotype suggests that a reversal of *MuDR(p5)* was occurring in these plants, but that it was incomplete. Supporting this hypothesis, after a second round of test crossing, these plants gave rise to an average of 49% heavily spotted progeny kernels (Figure 4A and Table 1). Together, these data suggest that *MuDR(p5)* eventually reactivated in all cases, but in some plants reactivation was delayed. In contrast, plants carrying only *MuDR(p4)* gave rise to a uniformly low frequency (5%) of very weakly spotted kernels, consistent with a more typically heritable silenced state. Thus, although both *MuDR(p4)* and *MuDR(p5)* had been exposed to *Muk* in a previous generation, *MuDR(p4)* remained silenced, whereas *MuDR(p5)* eventually reverted to an active state in all cases once *Muk* was lost.

**Table 1:** Activity of *MuDR(p5)* and *MuDR*(*p4*) in a family segregating for these elements and *Muk*.

Genotype a	Sample b	meth c	hm	weak	pale	T spot	total	% spot	% hm
P5 no *Muk* unmethylated d	1	no	97	0	93	97	190	51%	51%
	3	no	60	0	67	60	127	47%	47%
	total		157	0	160	157	317	50%	50%
P5 no *Muk* methylated e	10	yes	123	0	127	123	250	49%	49%
	18	yes	76	0	81	76	157	48%	48%
	total		199	0	208	199	407	49%	49%
P5 *Muk*	19	yes	17	14	25	31	56	55%	30%
	21	yes	36	38	92	74	166	45%	22%
	25	yes	36	50	181	86	267	32%	13%
	28	yes	12	14	66	26	92	28%	13%
	total		101	116	364	217	581	37%	17%
Both, no *Muk* unmethylated d	4	no	97	0	25	97	122	80%	80%
	5	no	131	0	33	131	164	80%	80%
	6	no	228	0	73	228	301	76%	76%
	7	no	144	0	43	144	187	77%	77%
	8	no	79	4	27	83	110	75%	72%
	9	no	107	0	34	107	141	76%	76%
	total		786	4	235	790	1025	77%	77%
Both, no *Muk* methylated e	2	yes	124	0	116	124	240	52%	52%
	22	yes	63	9	64	72	136	53%	46%
	26	yes	35	61	114	96	210	46%	17%
	total		222	70	294	292	586	50%	38%
Both *Muk*	14	yes	47	42	106	89	195	46%	24%
	15	yes	40	45	88	85	173	49%	23%
	total		87	87	194	174	368	47%	24%
P4 no *Muk*	29	yes	5	9	224	14	238	6%	2%
	34	yes	0	7	150	7	157	4%	0%
	total		5	16	374	21	395	5%	1%
P4 *Muk*	32	yes	0	0	192	0	192	0%	0%
	23	yes	3	6	252	9	261	3%	1%
	24	yes	3	2	38	5	43	12%	7%
	total		6	8	482	14	496	3%	1%
Neither	30		0	0	252	0	252	0%	0%
	31		0	0	182	0	182	0%	0%
	35		0	0	238	0	238	0%	0%
	36		0	0	273	0	273	0%	0%

a genotype of parents with respect to *MuDR(p5)* (P5), *MuDR(p4)* (P4) and *Mu killer* (*Muk*). Each parent plant was genotyped and then crossed to an *a1-mum2* tester and the resulting frequencies of heavy/medium (hm), weakly spotted (weak) and pale kernels were tabulated.

b parent plant numbers correspond to lane numbers in Figure 3.

c methylation status of *Mu1* in the parents of the families tabulated here, as determined by the blot in Figure 3C.

d parent plants had unmethylated *Mu1* and *MuDR* elements.

e parent plants had methylated *Mu1* and *MuDR* elements.

Plants carrying both *MuDR(p5)* and *Muk* also gave rise to a high frequency (37%) of spotted kernels, an average of 17% of which were heavily spotted (Figure 4A and Table 1). This ratio is consistent with a second generation of escape from *Muk*, where the progeny of these plants that carried *MuDR(p5)* but that lacked *Muk* had restored somatic activity. Thus, even after two successive generations of exposure to *Muk*, plants carrying *MuDR(p5)* retained the propensity to reactivate after *Muk* was lost. In contrast, lineages carrying only *MuDR(p4)* clearly lacked the propensity to reactivate even after only having been exposed to *Muk* for a single generation.

We also examined methylation at *MuDR* TIRs to see if the reversal of *Mu1* TIR methylation was associated with a reversal of methylation at *MuDR(p5)*. To do this, DNA that had been assayed for *Mu1* methylation (Figure 3C) was again digested with *Hin*fI, blotted and probed with a *MuDR* TIR fragment (Figure 4B). This analysis revealed that methylation at *MuDR(p5)* and *Mu1* correlated well.

**Figure 5:**
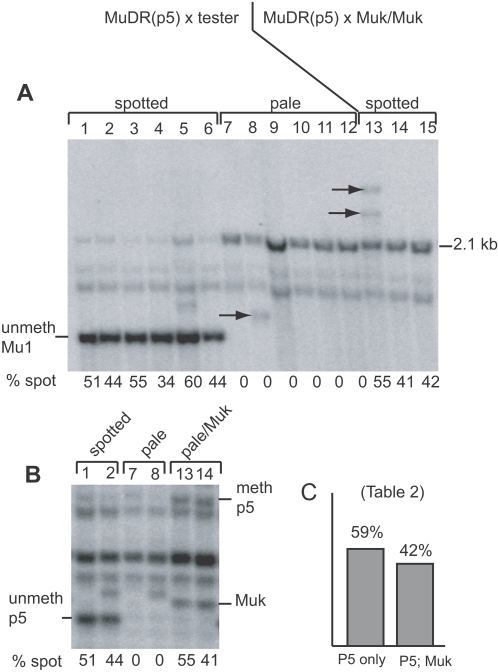
Genetic and Southern blot analysis of families segregating for *MuDR(p5)* and *Muk*. A) A *Hin*fI digest of two families probed with an internal portion of *Mu1*. The first was derived from a cross between a plant carrying an active *MuDR(p5)* element and an *a1-mum2* tester (lanes 1–12); the second was derived from a cross between the same plant carrying *MuDR(p5)* and a *Muk* homozygote. Methylated and unmethylated *Mu1* elements at *a1-mum2* are as indicated. Arrows indicate new insertions of Mu1 elements. B) DNA from representative individuals digested with *Hin*fI and probed with the *mudrA* TIR. Fragments resulting from methylated and unmethylated *Hin*fI sites within the TIR are as indicated, as is the fragment from *Muk*. Sample designations are the same as in panel A. C) Summarized frequencies of spotted kernels in progeny of test crosses of plants depicted in panel A.

It was important to show that reactivation of *MuDR(p5)* is a reproducible phenomenon. To do this, a single plant carrying reactivated *MuDR(p5)* was crossed to the *a1-mum2* tester and to a *Muk* homozygote. Kernels from the resulting families were grown, assayed for *Mu* element methylation, and test crossed. As expected, all of the plants from the *a1-mum2* test cross that inherited *MuDR(p5)* were unmethylated at *Mu1* (Figure 5A) and at *MuDR(p5)* (Figure 5B). All of these plants gave rise to approximately 50% spotted progeny kernels (Table 2). These data confirmed that *MuDR(p5)* remained active in a subsequent generation. In the family derived from the cross between the same *MuDR(p5)*-containing plant and a *Muk* homozygote, progeny plants contained methylated *Mu1* and *MuDR(p5)* (Figure 5A and B). Nevertheless, when these plants were test crossed, an average of 42% of the progeny kernels were spotted, indicating that *MuDR(p5)* had again escaped heritable silencing (Table 2). Plants that did not inherit *MuDR(p5)* did not give rise to any spotted progeny kernels, confirming that activity in this family was specific to *MuDR(p5)*.

**Table 2:** Recapitulation of Silencing and Reactivation of *MuDR(p5)* by *Muk*.

Genotype a	plant b	hm	weak	pale	T spot	total	%spot	%hm
P5 no *Muk*	1	64	0	62	64	126	51%	51%
	2	55	0	71	55	126	44%	44%
	3	69	0	57	69	126	55%	55%
	4	18	0	35	18	53	34%	34%
	5	72	52	83	124	207	60%	35%
	6	39	0	49	39	88	44%	44%
	total	460	53	357	513	870	59%	53%
no P5	7			216			0%	0%
	8			111			0%	0%
	9			11			0%	0%
	10			99			0%	0%
	11			119			0%	0%
	total			556			0%	0%
P5 with *Muk*	13	59	49	88	108	196	55%	30%
	14	34	22	80	56	136	41%	25%
	15	34	41	102	75	177	42%	19%
	16	38	35	110	73	183	40%	21%
	17	26	31	94	57	151	38%	17%
	18	23	32	83	55	138	40%	17%
	19	36	31	59	67	126	53%	29%
	20	7	17	153	24	177	14%	4%
	21	49	50	88	99	187	53%	26%
	total	306	308	857	614	1471	42%	21%
no P5	22			108			0%	0%
	23			54			0%	0%
	24			29			0%	0%
	25			64			0%	0%
	26			216			0%	0%
	27			196			0%	0%
	total			667			0%	0%

a genotype of parent plants with respect to *MuDR(p5)* (P5) and *Mu killer* (*Muk*). Each plant was crossed to an *a1-mum2* tester and the resulting frequencies of heavy/medium (hm), weakly spotted (weak) and pale kernels were tabulated.

b plant numbers 1–15 correspond to lane numbers in Figure 5.

### *MuDR(p4)* Reactivation Requires the Presence of an Active *MuDR(P5)* Element

**Figure 6:**
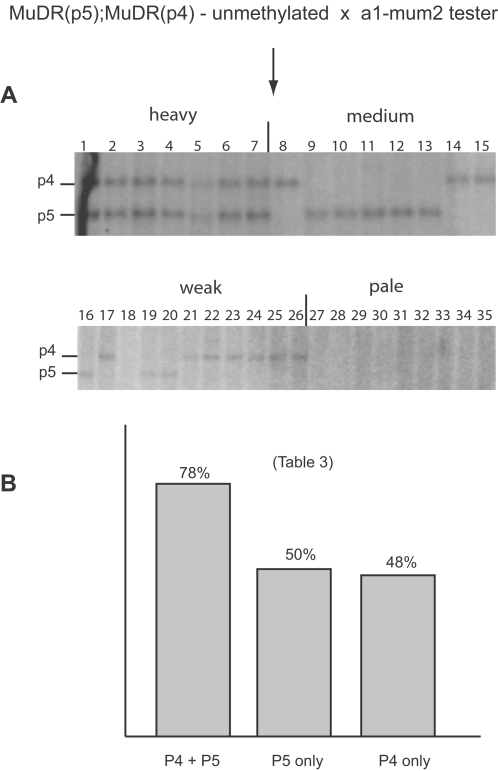
Genetic and Southern blot analysis of a family segregating for active *MuDR(p5)* and *MuDR(p4)* elements. A) *Xho*I digests of a family segregating for *MuDR(p5)* and *MuDR(p4)*, in which the female parent carried unmethylated *MuDR(p5)* and *MuDR(p4)* following the loss of *Muk*. Kernels were separated into classes based on somatic excision frequency, planted, and the resulting progeny plants were subjected to Southern blot analysis. B) Summarized frequency of spotted kernels in progeny of test crosses of the plants analyzed in panel A.

After the loss of *Muk*, *MuDR(p4)* never reactivated when it was by itself (Table 1). However, *MuDR(p4)* did become heritably reactivated in the presence of a reactivated *MuDR(p5)* element, but only when *MuDR(p5)* was fully active in the generation immediately following the loss of *Muk*. Plants that carried an active *MuDR(p5)* element (as judged by hypomethylation of *Hin*fI sites in both *MuDR(p5)* and *Mu1)*, also carried unmethylated *MuDR(p4)* elements (Figure 4B, lanes 7 and 8). When these plants were test crossed, they gave rise to an average of 77% spotted progeny, consistent with the independent segregation of two active *MuDR* elements (Table 1). To test this hypothesis, kernels from one of these families were planted and the resulting plants were subjected to Southern blot analysis (Figure 6A) and were test crossed (Table 3). In this family, both *MuDR(p5)* and *MuDR(p4)* cosegregated with *Mu* activity. All spotted kernels in this family carried either *MuDR(p5)*, *MuDR(p4)* or both, while none of the pale kernels had either. Plants carrying either *MuDR(p5)* or *MuDR(p4)* gave rise to an average of 50% and 48% spotted kernels respectively. Plants that carried both elements gave rise to an average of 78% spotted kernels, consistent with the independent assortment of two unlinked active *MuDR* elements (Table 3). Those that carried neither element did not give rise to any spotted kernels (data not shown). The elements also showed a positive dosage effect; the most heavily spotted kernels carried both elements (7/7) while the more moderate or weakly spotted kernels (19/19) carried a single *MuDR* element. These data demonstrate that both elements were active in this family, and that they were the only active elements present. Since *MuDR(p4)* alone never exhibited reactivation in this family (Table 1) or any other we have examined (see below), we suggest that *MuDR(p4)* required the presence of active *MuDR(p5)* to become reactivated.

**Table 3:** Activation of *MuDR(p4)* by *MuDR(p5)*.

genotype	plant a	spotted	pale	total	%spot
P4+P5	2	137	40	177	77%
	4	145	37	182	80%
	6	56	13	69	81%
	7	97	30	127	76%
	total	435	120	555	78%
P5 only	9	82	88	170	48%
	10	45	61	106	42%
	12	48	47	95	51%
	16	84	72	156	54%
	20	89	81	170	52%
	total	348	349	697	50%
P4 only	21	31	31	62	50%
	22	13	26	39	33%
	23	70	59	129	54%
	24	108	113	221	49%
	25	79	95	174	45%
	total	301	324	625	48%

a plant numbers correspond to lane numbers in Figure 6. Note that only a subset of the plants were test crossed.

**Figure 7:**
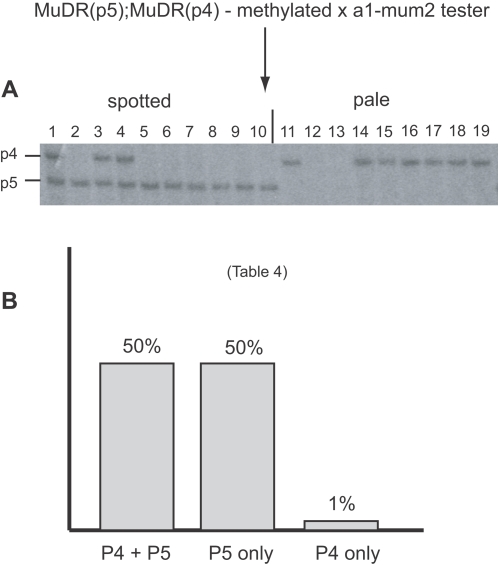
Genetic and Southern blot analysis of a family derived from a plant that carried *MuDR(p5)* and *MuDR(p4)* in which reactivation was delayed and both elements were still methylated in the first generation following the loss of *Muk*. A) *Xho*I digests of a family segregating for *MuDR(p5)* and *MuDR(p4)*, in which the female parent carried methylated *MuDR(p5)* and *MuDR(p4)*. Kernels were separated into classes based on somatic excision frequency, planted, and the resulting progeny plants were subjected to Southern blot analysis. B) Summarized frequency of spotted kernels in progeny of test crosses of plants depicted in panel A.

For comparison, we examined the heritable activity of *MuDR(p4)* in plants in which there had been a delay in *MuDR(p5)* reactivation. As described above, these plants carried methylated *MuDR* and *Mu1* TIRs in the generation immediately after the loss of *Muk* (Figures 3 and 4). However, when these plants were test crossed, they gave rise to an average of 50% heavily spotted progeny kernels (Table 1). One plant and its progeny were examined in detail. In this plant, both *MuDR(p5)* and *MuDR(p4)* had remained at least partially inactive in the first generation after the loss of *Muk* (Figure 4B, lanes 9 and 10, and 4D). Despite having two potentially active elements, this and all similar families segregated only 50% spotted progeny kernels, as if only one of these two *MuDR* elements had become reactivated in this generation (Table 1). Southern blot analysis of progeny of this plant revealed that *MuDR(p5)*, but not *MuDR(p4)*, co-segregated with activity (Figure 7A). All the plants grown from spotted kernels in this family carried *MuDR(p5)*, but the presence or absence of *MuDR(p4)* had no effect on activity; three of ten plants grown from spotted kernels carried *MuDR(p4)*, as did seven of nine plants grown from pale kernels. This experiment demonstrated that *MuDR(p4)* was not active in this family. It also showed that in this generation, an active *MuDR(p5)* element had no influence on the heritable activity of *MuDR(p4)*. Plants that carried both *MuDR(p5)* and *MuDR(p4)*, when test crossed, gave rise to only 50% spotted progeny (Table 4 and Figure 7B). Together, these results suggest that *MuDR(p4)* could be responsive to a reactivated *MuDR(p5)*, but only in the generation immediately following the loss of *Muk*.

**Table 4:** Lack of heritable reactivation of *MuDR(4)*.

genotype	plant a	spotted	pale	total	%spot
P5 only	2	89	90	179	50%
	5	65	67	132	49%
	6	82	83	165	50%
	8	62	57	119	52%
	9	101	101	202	50%
	10	47	47	94	50%
	total	446	445	891	50%
P5+P4	1	91	101	192	47%
	3	57	59	116	49%
	4	49	39	88	56%
	total	197	199	396	50%
P4 only	11	0	145	145	0%
	14	0	120	120	0%
	15	5	104	109	5%
	16	0	57	57	0%
	17	0	145	145	0%
	18	6	82	88	7%
	19	0	103	103	0%
	total	11	756	767	1%
neither	12	0	95	95	0%
	13	0	98	98	0%
	total	0	193	193	0%

a plant numbers correspond to lane numbers in Figure 7.

It is unclear as to precisely when *MuDR(p5)* must be active in order to alter the trajectory of *MuDR(p4)* silencing. Only those plants that showed hypomethylation at *MuDR* and *Mu1* TIRs that were grown from more heavily spotted kernels gave rise to progeny with active *MuDR(p4)* elements. This suggests an active *MuDR(p5)* element was required quite early in development in order to reactivate *MuDR(p4)*. The aleurone and the mature plant are the result of a double fertilization event. One sperm fuses with the egg cell of the female gametophyte to form the embryo. The second fuses to the diploid central cell to give rise to the triploid endosperm. The egg cell and the central cell are derived from a post-meiotic mitotic division in the female gametophyte. With that in mind, it is interesting to note that eight of ten heavily and medium spotted kernels gave rise to plants with hypomethylated *Mu* elements. In contrast, none of the plants grown from more weakly spotted kernels gave rise to plants with hypomethylated *Mu* elements. The fact that the methylation status of *MuDR(p5)* in the mature plant correlated so well with the phenotype of the kernels suggests that *MuDR(p5)* reactivation that was not delayed most often occurred prior to the post-meiotic mitotic cell division. Together, these data suggest that the window of opportunity for activation of *MuDR(p4)* by *MuDR(p5)* may be a very narrow one. Indeed, it may be restricted to the gametophyte, or even meiosis II.

### All Aspects of *MuDR* Activity Are Restored Following Reactivation

Although somatic excision of a reporter element is a reliable indicator of *Mu* activity, it only represents one aspect of that activity, which only requires MURA transposase function [26],[27]. Insertional activity, either of the reporter element or of *MuDR* itself requires both *mudrA* and *mudrB* expression. The analysis portrayed in Figure 5 demonstrated that a reactivated *MuDR(p5)* element could cause new insertions of *Mu1*. When *Mu1* is methylated, the size of the fragments following digestion varies depending on the position of the element. The element at *a1-mum2* is 2.1 kb. Other sizes present in single individuals represent independent new insertions of *Mu1*. The presence of new *Mu1* fragments in progeny of plants that carried reactivated *MuDR(p5)* (Figure 5A, lanes 8 and 13) indicates that this element can cause new insertions of *Mu1*, consistent with reactivation of both *mudrA* and *mudrB* functions.

We also examined the propensity of reactivated *MuDR(p5)* and *MuDR(4)* to duplicate themselves by test crossing a series of individuals that carried active versions of either *MuDR(p5)* or *MuDR(p4)*. In each case, the plants were derived from a family that had segregated genetically for a single active *MuDR* element. In the absence of new duplications of these *MuDR* elements, the expectation would be that each resulting family would also segregate 50% spotted progeny kernels. Ratios significantly higher than 50% are the result of *MuDR* duplication events [21]. The frequency of ears showing ratios of spotted kernels significantly greater than 50% provides an estimate of the duplication frequency, which we have shown can vary from position to position [24]. Of 100 ears derived from plants carrying silenced *MuDR(p5)* in the presence of *Muk*, none had ratios significantly greater than 50% spotted kernels (data not shown), indicating that *MuDR(p5)* does not transpose in the presence of *Muk*. In contrast, following reactivation we found that both *MuDR(p4)* and *MuDR(p5)* were competent to transpose at a frequency of 10% for *MuDR(p5)* and 18% for *MuDR(p4)* (data not shown). These data demonstrate that although both somatic and transpositional activity of *MuDR(p5)* is repressed in the presence of *Muk*, both manifestations of activity are restored once *Muk* is lost via genetic segregation.

We also wanted to confirm that the “rescue” of *MuDR(p4)* in the previous experiment was due to the presence of a reactivated *MuDR(p5)* element. To test the effects of *Muk* on *MuDR(p4)* in the absence of *MuDR(p5)*, plants carrying active *MuDR(p4)* were crossed to *Muk* heterozygotes, and the resulting plants were then test crossed (Figure 1). As before, unlike *MuDR(p5)*, which showed clear evidence of reactivation following the loss of *Muk*, *MuDR(p4)* remained heritably silenced (Table 5). Thus, *MuDR(p4)* in the absence of an active *MuDR(p5)* element showed a typical pattern of heritable silencing after being exposed to *Muk*.

**Table 5:** Heritable silencing of *MuDR(p4)* by *Muk*.

Genotype	plant	spotted	pale	total	%spot
P4 no *Muk*	1	87	116	203	42.9%
	2	186	174	360	51.7%
	3	89	87	176	50.6%
	4	69	51	120	57.5%
	5	65	33	98	66.3%
	6	44	44	88	50.0%
	7	112	151	263	42.6%
	8	79	76	155	51.0%
	total	731	732	1463	50.0%
P4 with *Muk*	1	5	338	343	1.5%
	2	1	308	309	0.3%
	3	20	252	272	7.4%
	4	15	252	267	5.6%
	5	5	112	117	4.3%
total		46	1262	1308	3.5%

**Figure 8:**
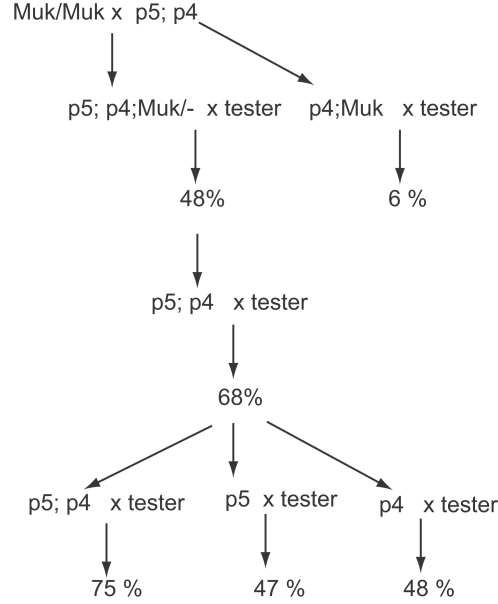
A graphic representation of a lineage in which a plant carrying active *MuDR(p5)* and *MuDR(p4)* was crossed to a *Muk* homozygote, and resulting progeny plants were subsequently test crossed. Percent figures refer to the summarized frequency of spotted progeny kernels derived from each cross.

In order to replicate the “rescue experiment”, a plant carrying active *MuDR(p5)* and *MuDR(p4)* elements was crossed to a *Muk* homozygote. Progeny plants were genotyped and test crossed (Figure 8). Plants that carried only *MuDR(p4)* and *Muk* gave an average ratio of spotted kernels of 6%, consistent with our previous result that *MuDR(p4)* without *MuDR(p5)* is heritably silenced by *Muk*. Plants that carried *Muk* with *MuDR(p5)* alone or with *MuDR(p4)* gave an average frequency of spotted progeny of 48%, consistent with reactivation of *MuDR(p5)* following the loss of *Muk*. Progeny of this cross that carried both *MuDR(p5)* and *MuDR(p4)* but that lacked *Muk* were test crossed again. One individual gave rise to a ratio of spotted kernels of 68%, consistent with the independent segregation of two active elements. In the next generation, somatic activity segregated with both elements; plants carrying both *MuDR(p4)* and *MuDR(p5)* gave rise to a 75% ratio, and those with either *MuDR(p4)* or *MuDR(p5)* by itself gave rise to roughly 50% ratios (Table 6). These data strongly support the hypothesis that, although *MuDR(p4)* is invariably silenced in the absence of *MuDR(p5)*, a reactivated *MuDR(p5)* element can cause *MuDR(p4)* to reactivate as well.

**Table 6:** Recapitulation of *MuDR(p4)* reactivation with *MuDR(p5)*.

Genotype	plant	spotted	pale	total	% spot
P5	1	102	111	213	47.9%
	2	105	107	212	49.5%
	3	137	188	325	41.1%
	4	80	70	160	56.3%
	5	59	60	119	49.6%
	6	46	49	95	48.4%
	7	79	100	179	44.1%
	8	65	76	141	46.1%
	total	673	761	1444	47.3%
P4	9	91	107	210	49.0%
	10	69	63	132	52.3%
	11	74	106	189	43.9%
	total	234	276	531	48.0%
P5+P4	12	57	22	81	72.8%
	13	150	44	201	78.1%
	14	152	53	206	74.3%
	15	79	34	115	70.4%
	total	438	153	603	74.6%

### A Duplicate Copy of *MuDR(p5)* Remains Inactive Following Exposure to *Muk*

**Figure 9:**
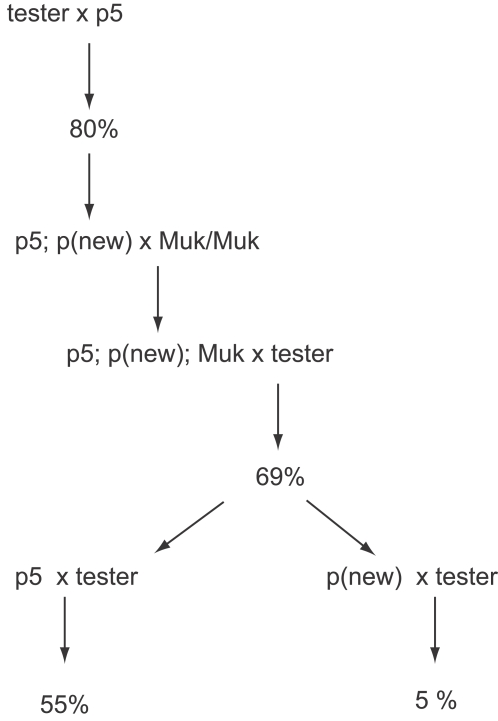
A graphic representation of a lineage in which *MuDR(p5)* and a duplicate copy of that element were crossed to a *Muk* heterozygote. Percent figures refer to the summarized frequency of spotted progeny kernels derived from each cross.

If the reactivation effect we observe for *MuDR(p5)* were a function of position, then we would expect that, if this element transposed to a new position, it would exhibit a more typical heritable response to *Muk*. To test this hypothesis, plants carrying *MuDR(p5)*, a transposed copy of this element at a second unlinked position and *Muk* were test crossed (Figure 9). Resulting progeny plants grown from spotted kernels were genotyped for *MuDR(p5)* and *Muk* and test crossed a second time (Table 7). Plants carrying *MuDR(p5)* that lacked *Muk* gave rise to ears that segregated for one or more active *MuDR* elements and averaged 55% spotted progeny kernels. In contrast, siblings that inherited only the second *MuDR* element and not *MuDR(p5)* gave rise to a much lower frequency of spotted kernels (5%), consistent with the kind of heritable silencing that is typical for *MuDR* elements after having been exposed to *Muk*. These results suggest that, while *MuDR(p5)* reactivates once *Muk* is segregated away, the duplicate copy of this element remained heritably silenced. These data strongly suggest that the reduction of heritable silencing at *MuDR(p5)* is a function of chromosomal position and not sequence, since this effect can be reversed following transposition.

**Table 7:** A transposed copy of *MuDR(p5)* is heritably silenced by *Muk*.

Genotype a	plant	spotted	Pale	Total	% Spot
P5	1	31	30	61	50.8%
	2	110	107	217	50.7%
	3	60	63	123	48.8%
	5	167	76	243	68.7%
	6	54	61	115	47.0%
	7	115	47	162	71.0%
	8	99	133	232	42.7%
	9	90	26	116	77.6%
	10	89	53	142	62.7%
	11	60	59	119	50.4%
	12	62	233	295	21.0%
	13	98	30	128	76.6%
	14	115	79	194	59.3%
	15	94	100	194	48.5%
	16	151	52	203	74.4%
	17	119	127	246	48.4%
	18	78	34	112	69.6%
	total	1751	1453	3204	54.7%
P(new)	19	0	210	210	0.0%
	20	53	112	165	32.1%
	21	6	65	71	8.5%
	22	7	195	202	3.5%
	23	4	72	76	5.3%
	24	7	203	210	3.3%
	25	0	216	216	0.0%
	26	0	42	42	0.0%
	27	0	30	30	0.0%
	28	0	273	273	0.0%
	total	77	1418	1495	5.2%

a all plants lacked *Muk*. Plants either carried *MuDR(p5)* or they lacked that element but carried a second element (P(new)).

### *MuDR(p5)* Is Inserted into the 5′ UTR of a Conserved Gene Near a GA-Rich Sequence

In order to determine the local chromosomal environment around *MuDR(p5)* and *MuDR(p4)*, inverse PCR was used to clone sequences flanking the insertions. DNA from plants carrying either element was digested with *Xho*I, which gives rise to fragments of 2.6 and 2.4 kb corresponding to *MuDR(p4)* and *MuDR(p5)* respectively (Figure 3B). The DNA was then ligated and primers specific to *MuDR* were used on the circularized fragments to amplify fragments of the expected sizes (see Materials and Methods for details). The products were sequenced on both strands, and the resulting sequences were used to design flanking primers. These primers were then used with *MuDR*-specific primers on DNA from families segregating for *MuDR(p4)* or *MuDR(p5)*. In each case, these primer pairs specifically amplified a product only in samples containing the *MuDR* elements (data not shown). Sequences were extended using publicly available maize genomic sequences, and these sequences were used to design primers matching DNA sequences present to the other side of each element. Nine base pair target site duplications, a characteristic feature of *Mu* insertions, were identified in each case. Further, *Xho*I and *Hin*fI sites in the flanking sequences obtained from the public databases also correlated well with data obtained from Southern blot restriction data.

**Figure 10:**
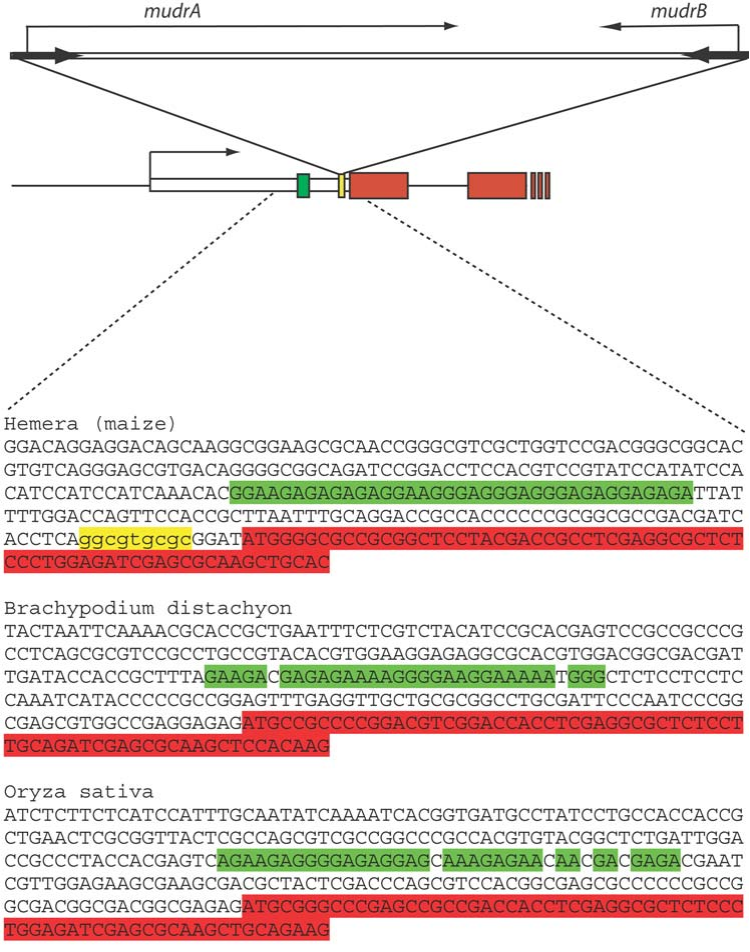
A representation of the region into which *MuDR(p5)* is inserted. Sequences in yellow represent the target site duplication that was produced upon insertion. Sequences in green are the GA-rich sequences identified near the insertion. Sequences in red are presumed coding sequences. The rice homolog is the gene that best matches the *Hemera* gene in maize.

Given its propensity to reactivate, we were particularly interested in sequences flanking *MuDR(p5)*. This element was inserted into the 5′ UTR just 4 base pairs proximal to the start codon of a putative ORF of unknown function (Figure 10), which we designate here *Hemera*, after the Greek goddess of the day, who was believed to disperse the night's mist each morning. Genes homologous to *Hemera* can be detected other grasses such as rice and *Brachypodium distachyon*. This conservation, along with the presence cDNA sequences in the database from several species, including maize, suggests that this gene is functional. The insertion of *MuDR(p5)* was 69 bp downstream of a 37 bp GA-rich sequence composed largely of GA repeats. Interestingly, although the rice and *B. distachyon* 5′ UTRs are not homologous to the maize sequence by sequence similarity, each of them has a GA-rich sequence roughly the same distance from the putative start of translation. These data suggest that sequence composition, rather than sequence order, may be conserved at this gene in these three species. Homologues of *Hemera* are also present in dicots, including papaya, grape, *Arabidopsis* and poplar. Although some of these sequences carry GA or TC rich regions near the putative start of translation, their positions are not conserved between species (Figure S1).

*MuDR(p4)* was also inserted into a conserved gene of unknown function (Figure S2). Based on a comparison with cDNAs from several species, it appears that the insertion is into an intron, 401 bp upstream of the putative start of translation. Interestingly, the 5′ portion of this intron contains a region rich in TCs, as does the 5′ portion of the paralogous rice gene, which contains a long GA-rich sequence. Since *MuDR(p4)* does not reactivate following exposure to *Muk*, these data suggest GA/TC-rich sequences by themselves are not sufficient to permit reactivation. However, it is possible the presence of these sequences near *MuDR(p4)* make it particularly responsive to active *MuDR(p5)*. Analysis of additional positions, and combinations of positions will be informative, but an unambiguous demonstration of the propensity for *cis*-acting sequences will require mutation of those sequences in a transgenic context.

## Discussion

The experiments described here detail a position effect that alters the heritability of the silenced state of a maize transposon. The experiments were possible because of the absence of spontaneous *Mu* transposon silencing in our lines and the availability of a single silencing locus (*Muk*) that can reliably and heritably silence *MuDR* elements. Because heritability is the rule for *MuDR* silencing by *Muk*, it was possible to screen for exceptions to this rule in order to uncover variation in the ability of chromosomal positions to maintain silencing over multiple generations. One such exception is *MuDR(p5)*, which fails to maintain silencing. The fact that a transposed copy of *MuDR(p5)* showed a more typically heritable pattern of *Muk*-induced silencing demonstrates that *MuDR(p5)* is exceptional because of its position rather than its sequence.

To our knowledge, this is the only known example of a specific locus competent to reverse epigenetic silencing of flanking sequences. In plants, a related (albeit reversed) phenomenon can be found at the FLC locus in *Arabidopsis thaliana*. In that case, the FLC gene is apparently competent to alter the activity of neighboring genes via an epigenetically mediated pathway [28]. When a T-DNA encoded resistance gene is integrated near the FLC gene, its expression is down regulated in response to cold temperatures, and this down-regulation is dependent at least in part on components of the small RNA mediated silencing pathway. The difference is that FLC attracts factors that down-regulate gene expression, and *Hemera* apparently attracts factors that reverse silencing. Nevertheless, in each case it appears that there are *cis*-acting sequences that can alter the epigenetic state of inserted genes.

Interestingly, the kind of epigenetic resetting we see with *MuDR(p5)* is typical in animals, although the role of position remains poorly understood. In certain cell types at certain times, massive changes in patterns of histone and DNA methylation are observed. This process, which is thought to be required for the elimination of some epigenetic marks and their replacement with others, is particularly pronounced in the pre-implantation embryo of mammals [29]. The same is true of primordial germ cells, where this process of epigenetic reprogramming is thought to be involved in the restoration of totipotency [30]. In mammals, exceptional instances in which DNA methylation is *not* lost, are associated with imprinted genes and deeply silenced transposons [31]. In some cases it has been shown that a close association between transposon and host gene can lead to heritable changes in phenotype. For instance, the *Agouti viable yellow* (*A(vy)*) locus in mice is under the control of an IAP retrotransposon. Hypomethylation of this element results in expression of the gene and yellow coat color. Epigenetic variants of this allele can be transmitted from generation to generation, and it is hypothesized that the heritable epigenetic effects of on *A(vy)* are due to a failure to remove epigenetic marks due to the close association of the IAP element with the coding sequence [32].

In *Drosophila*, changes in the efficiency of epigenetic resetting can have important consequences. A hyperactive version of a JAK kinase, *hop^Tum-1^*, causes tumor formation. It does so because counteracts heterochromatic gene silencing, which is an important regulatory pathway for tumor suppression [33],[34]. Enhancers of the *hop^Tum-1^* allele included several components of the heterochromatin formation pathway, including HP1 and several Suppressors of variegation mutations, which were first identified due to their effects on position effect variegation. Remarkably, not only can *hop^Tum-1^* cause tumors in one generation, but it can increase the propensity for the wild-type offspring of mutant flies to have tumors as well [35]. It is hypothesized that the *hop^Tum-1^* mutation antagonizes the normal process by which epigenetic states are reset each generation by allowing genes that should be heritably silenced to take on a heritably active state.

Plants are distinct from animals in the sense that they lack a dedicated germ line. Instead, somatic meristem tissue differentiates into germinal cells each generation. A wealth of information suggests that the result of this difference is that epigenetic changes in plants are more readily transmitted from generation to generation [36]. Nevertheless, it is likely that in plants, as in animals, at least a subset of genes in are reset each generation order to ensure that the epigenetic state of each embryo is roughly equivalent. DNA methylation, for instance, increases in the meristem as it ages, and these changes must presumably be reversed each generation [37],[38]. We suggest that *Hemera* may represent a gene whose epigenetic state must be reset each generation. If *Hemera* were epigenetically silenced in the floral meristem and upregulated in the embryo, for instance, then perhaps that epigenetic regulation must be relieved during or following meiosis. It will be interesting to see if differences in expression levels of *Hemera* correlate with changes in chromatin configuration or DNA methylation, and whether or not these changes correlate with changes at *MuDR(p5)*.

It should be emphasized that the variation we observe is not in the propensity to become silenced; *MuDR(p5)* is effectively silenced by *Muk*. Given that *MuDR(p5)* TIR sites are methylated at the *Hin*fI site, it is also unlikely that this element is exclusively inactivated at the post-transcriptional level. Rather, the effect we observed appears to be specifically associated with the efficiency with which transcriptional silencing of this element is heritably propagated in the absence of the trigger. The loss of methylation at *MuDR(p5)* may not be a passive process; our assay for methylation, a *Hin*fI digest, depends on methylation of a CG site. Since CG methylation can be maintained passively through the activity of maintenance methyl-transferases such as MET1, the loss of methylation at this site may reflect an active de-methylation process. Active demethylation has been observed as a consequence of DNA glycosylase activity in plants, and is often associated with repetitive elements such as transposons [39],[40]. It will be interesting to see whether or not the reversal of methylation we see at *MuDR(p5)* is due to similar activity in maize. It will be particularly interesting if mutations of maize DNA glycosylase genes affect *MuDR(p5)* reactivation.

We do not know the cause of the position effect on *MuDR(p5)*. The fact that this element is inserted into an expressed portion of a gene may have been sufficient to reverse silencing, but *Mu* elements often insert into or near genes and nearly all *MuDR* elements are silenced when high copy number *Mu* lines are crossed to *Muk* [22]. The presence of GA repeats near the insertion is intriguing, as GA repeats have been associated with programmatic changes in chromatin structure and in particular with the active replacement of histones [9]. Although we have not established that this is the case at *MuDR(p5)*, we do note that the rice and *B. distachyon* homologs of *Hemera* also have GA-rich sequences just upstream of the start of the ORFs. Although the sequence of the GA-rich regions in the maize, rice and *B. distachyon* genes are not similar in sequence, they do have similar sequence composition (100%, 89% and 96% GA respectively). These blocks of sequences are roughly the same distance from the first ATG of each gene, 82 bp, 83 bp and 89 bp for maize, rice and *B. distachyon* respectively. Given the phylogenetic distance between these species (roughly 50 million years [41]), the conserved positioning of these blocks at the same distance from the start of translation in each gene suggests that they may have a conserved function.

In addition to the position effects we observed, our data also suggests that epigenetically determined states of competency can change over time. Specifically, we provided evidence that a silenced *MuDR(p4)* element could respond to a reactivated *MuDR(p5)* element, but only for a brief period of time. This was revealed because of variations in the rate at which *MuDR(p5)* became reactivated. In some cases, it was immediately after the loss of *Muk*, as evidenced by the high level of somatic activity in the aleurone and the complete loss of methylation in the growing F2 plants (Figures 3 and 4). In these cases, when *MuDR(p4)* was also present, it too was reactivated. However, in those cases in which *MuDR(p5)* reactivation was delayed (weakly spotted kernels, variegated *a1-mum2* suppression and TIR methylation), *MuDR(p4)* was not reactivated. In the subsequent generation, even though *MuDR(p5)* had become fully reactivated, it had no effect on a previously silenced *MuDR(p4)*. We hypothesize that silencing of *MuDR* elements is a progressive process that involves successively deeper silenced states, from responsive to a second, active element, to refractive to that element. Thus, immediately after *Muk* was lost due to genetic segregation, *MuDR(p4)* silencing was not completely established, and so this element was responsive to active *MuDR(p5)*. After a round of meiosis, *MuDR(p4)* had become fully refractive to *MuDR(p5)*. Perhaps passage through meiosis of a previously silenced transposon acts as a check-point, during which provisionally established silenced states are made more permanent. If our interpretation of the data is correct, then the epigenetic state of *MuDR(p4)* can change over time, even once the silencing trigger (*Muk*) has been lost. This is consistent with what we know about silencing mechanisms in plants, in which chromatin remodeling factors, DNA methylation and siRNAs form a self-reinforcing loop [42]. *MuDR(p4)* silencing may represent an illustration of how this process can deepen a silent state over time, resulting in a shift from competency to respond to a second, active element to a refractive state in the course of a generation. Similarly but conversely, *MuDR(p5)* may represent a process by which silenced states can be reversed over time through the activity of cis-acting factors. The delay in *MuDR(p5)* reactivation in many of the plants examined suggests that reactivation, like silencing, can be a progressive process. Our data suggest that even after a trigger is lost, a series of additional and progressive changes can continue to occur. This is perhaps the most fascinating aspect of epigenetic modifications: time matters. Changes triggered in one generation can manifest themselves over multiple subsequent generations.

Historically, an emphasis has been on mechanisms by which epigenetic information is propagated from generation to generation, a classic example being paramutation [43]. Our data suggest that an equally important process may be the erasure of epigenetic modifications that have occurred in plants in the meristem prior to meiosis. The cis-acting factors that appear to be responsible for reversing *MuDR(p5)* silencing may provide an important clue concerning the mechanism of this erasure.

## Supporting Information

Figure S1 A representation of the region immediately upstream of the putative start of translation of homologs of *Hemera* in poplar (two paralogs), papaya, *Arabidopsis*, and grape. Sequences in green are the GA-rich sequences identified near the insertion. Sequences in blue are TC-rich sequences. Sequences in red are presumed coding sequences.
(0.53 MB EPS)

Figure S2 A representation of the region immediately upstream of the putative start of translation of the gene into which *MuDR(p4)* is inserted, and the homolog of that gene in rice. Sequences in green are the GA-rich sequences identified near the insertion. Sequences in blue are TC-rich sequences. Sequences in red are presumed coding sequences.
(0.56 MB EPS)
