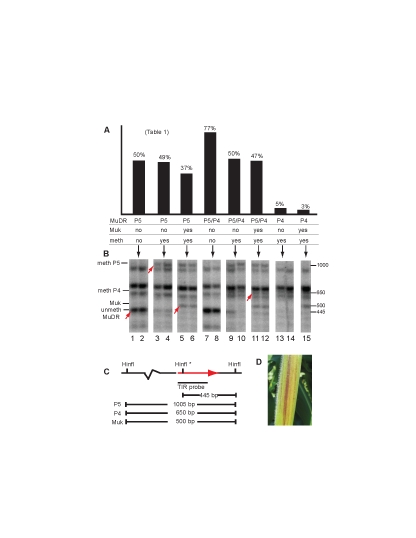# Correction: A Position Effect on the Heritability of Epigenetic Silencing

**DOI:** 10.1371/annotation/dd58c52f-161d-4a3f-b560-c9fd78307f85

**Published:** 2009-02-13

**Authors:** Jaswinder Singh, Michael Freeling, Damon Lisch

In place of Figure 4, Figure 6 was mistakenly duplicated. The correct Figure 4 is available here: 

**Figure:**